# The Role of Tenascin C in Cardiac Reverse Remodeling Following Banding–Debanding of the Ascending Aorta

**DOI:** 10.3390/ijms22042023

**Published:** 2021-02-18

**Authors:** Mireia Perera-Gonzalez, Attila Kiss, Philipp Kaiser, Michael Holzweber, Felix Nagel, Simon Watzinger, Eylem Acar, Petra Lujza Szabo, Inês Fonseca Gonçalves, Lukas Weber, Patrick Michael Pilz, Lubos Budinsky, Thomas Helbich, Bruno Karl Podesser

**Affiliations:** 1 Ludwig Boltzmann Institute for Cardiovascular Research at the Center for Biomedical Research, Medical University of Vienna, 1090 Vienna, Austria; mireia.p.gonzalez@gmail.com (M.P.-G.); attila.kiss@meduniwien.ac.at (A.K.); phk.philippkaiser@gmail.com (P.K.); michael.holzweber@gmail.com (M.H.); Felix.Nagel@cardio.lbg.ac.at (F.N.); watzinger.simon@hotmail.com (S.W.); eylem-@hotmail.com (E.A.); petra.szabo@meduniwien.ac.at (P.L.S.); goncalvesf.ines@gmail.com (I.F.G.); lukas.weber@meduniwien.ac.at (L.W.); patrick.pilz@meduniwien.ac.at (P.M.P.); 2 Bioengineering and Aerospace Engineering Department, Carlos III University of Madrid, 28911 Madrid, Spain; 3 Department of Cardiac Surgery, University Hospital St. Poelten, 3100 St. Poelten, Austria; 4 Preclinical Imaging Lab at the Center of Biomedical Research, Department of Radiology, Medical University of Vienna, 1090 Vienna, Austria; lubos.budinsky@meduniwien.ac.at (L.B.); thomas.helbich@meduniwien.ac.at (T.H.)

**Keywords:** reverse remodeling, tenascin C, angiotensin-converting enzyme, cardiac magnetic resonance imaging

## Abstract

Background: Tenascin-C (TN-C) plays a maladaptive role in left ventricular (LV) hypertrophy following pressure overload. However, the role of TN-C in LV regression following mechanical unloading is unknown. Methods: LV hypertrophy was induced by transverse aortic constriction for 10 weeks followed by debanding for 2 weeks in wild type (Wt) and TN-C knockout (TN-C KO) mice. Cardiac function was assessed by serial magnetic resonance imaging. The expression of fibrotic markers and drivers (angiotensin-converting enzyme-1, ACE-1) was determined in LV tissue as well as human cardiac fibroblasts (HCFs) after TN-C treatment. Results: Chronic pressure overload resulted in a significant decline in cardiac function associated with LV dilation as well as upregulation of TN-C, collagen 1 (Col 1), and ACE-1 in Wt as compared to TN-C KO mice. Reverse remodeling in Wt mice partially improved cardiac function and fibrotic marker expression; however, TN-C protein expression remained unchanged. In HCF, TN-C strongly induced the upregulation of ACE 1 and Col 1. Conclusions: Pressure overload, when lasting long enough to induce HF, has less potential for reverse remodeling in mice. This may be due to significant upregulation of TN-C expression, which stimulates ACE 1, Col 1, and alpha-smooth muscle actin (α-SMA) upregulation in fibroblasts. Consequently, addressing TN-C in LV hypertrophy might open a new window for future therapeutics.

## 1. Introduction

Left ventricular hypertrophy (LVH) is an adaptive mechanism of the heart to compensate for pressure overload. This was described in a landmark clinical paper by William Grossmann in 1975 [1]. The effects of ventricular unloading have been discussed mainly in patients undergoing aortic valve replacement for almost as long [2]. One study suggested that mechanical reduction of left ventricular (LV) afterload significantly reduced LV wall stress [3], in a mechanism described by Levin et al. [4] as “reverse remodeling”. This is associated with an improvement in LV ejection fraction (LVEF%) and a reduction in left ventricular mass (LVM). At the cellular level, this phenomenon involves the functional improvement of cardiomyocytes and partial reduction of extracellular matrix (ECM) deposition [5]. Clinical data are diverse depending on the clinical population. Whereas patients with preserved ejection fraction benefit from unloading, patients with heart failure do not show the desired regression but a further increase in fibrosis [6]. Animal models have the unique benefit of providing a perfect environment, thereby reducing confounding factors such as age, duration of disease, etc. In an elegant study in mice, Bjornstad et al. [7] recently demonstrated that after 4 weeks of banding and consecutive debanding (14 days) a progressive improvement in the function and regression of myocardial mRNA levels of brain natriuretic peptide (BNP), atrial natriuretic protein (ANP), α-smooth muscle actin, and β-myosin heavy chain was observed. However, changes in ECM component expression and their role in cardiac function upon reverse remodeling still remain largely unknown. Tenascin-C (TN-C) is a glycoprotein of the ECM expressed during embryogenesis, tumorigenesis, and vasculogenesis, as well as in diseases such as myocardial infarction, LVH, and myocarditis [8]. Members of the tenascin protein family are known as “talented proteins in search of functions” [9]. We have recently highlighted the role of TN-C in regulating interstitial fibrosis and myocardial function in response to pressure overload, demonstrating that high levels of TN-C were associated with adverse LV remodeling [10]. In addition, our recent study demonstrated that TN-C upregulation was associated with angiotensin-converting enzyme-1 (ACE-1) activity enhancement, suggesting the molecular interaction between ACE-1 and TN-C [11]. However, knowledge on the impact of TN-C on the ECM changes associated with reverse remodeling and cardiac function still remains elusive. Despite previous observations on LV morphometry of banded hearts, differences in progression of LVH and remodeling remain unassessed [12]. In the present study, transverse aortic constriction (TAC) was performed over 10 weeks to simulate pressure overload and the consecutive development of heart failure. This pressure-overload period was followed by a debanding operation and a two-week follow up period. Cardiac MRI was performed in cohorts of control (without TAC), banded (AJ and TN-C knockout (TN-C KO), and de-banded (AJ and TN-C KO) mice. 

## 2. Results

### 2.1. Experimental Groups

Left ventricular hypertrophy was induced by transverse aortic constriction (TAC) for 10 weeks in TN-C KO mice (*n* = 6) and in wild-type AJ (*n* = 8, Wild-type, Wt) mice. After 10 weeks, banded animals underwent overload relief by an aortic debanding surgery (*n* = 3–5/group). Sham-operated mice (*n* = 5) served as controls (Wt AJ). 

Table 1 describes animal characteristic of the experimental groups. Body weight (BW, in grams) and the heart weight-to-body weight ratios were significantly higher between control and Wt 10 mice (*p* < 0.001), indicating hypertrophy. In contrast, banding did not affect the above-mentioned parameters of hypertrophy in TN-C KO mice at 10 weeks (controls vs. TN-C KO 10). Debanding resulted in a significant reduction in both heart weight and heart weight-to-body weight ratio in Wt 12 (*p* < 0.05) whereas this regression was less pronounced in TN-C KO 12. These observations were paralleled by the LVM (in grams) and LVM/BW ratio, which was significantly higher in the Wt 10 group as compared to controls (*p* < 0.001), indicating hypertrophy. Debanding resulted in significant regression of LVM in Wt 12 (*p* < 0.001) whilst the regression was less pronounced in TN-C KO 12. Wet lung data were analyzed via the lung weight-to-body weight ratio (LW/BW) and showed pulmonary congestion (*p* < 0.01), which was markedly improved by mechanical unloading (*p* = 0.07).

### 2.2. Functional and Morphological Changes of Left and Right Ventricular Function Following Aortic Constriction and Debanding in Mice

**Figure 1:**
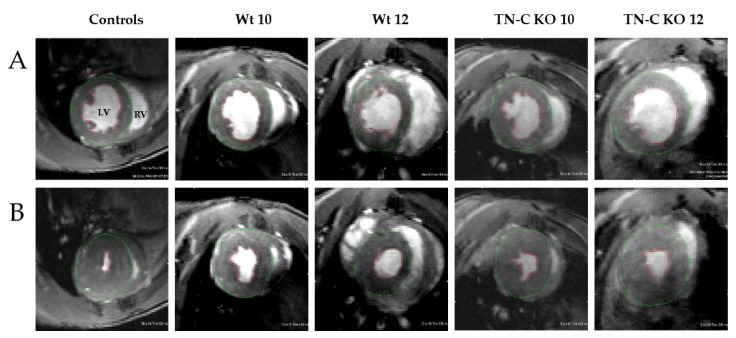
Comparison of short-axis MRI of an arbitrary mouse from each group (controls, Wt 10, Wt 12, TN-C KO 10, TN-C KO 12) in the end-diastole (**A**) and end-systole (**B**) phases of the cardiac cycle. Left ventricular (LV) and right ventricular (RV) regions are indicated in the top-left image as an example; the LV endocardium is outlined in red and the LV epicardium is outlined in green. Wt: Wild type and TN-C KO: Tenascin-C knockout.

Magnetic resonance imaging (MRI) was performed for all experimental study groups (controls, Wt, and TN-C KO). Figure 1 shows representative images of short-axis MRI of an arbitrary mouse from each group (controls, Wt 10, Wt 12, TN-C KO 10, TN-C KO 12) in the end-diastole (Figure 1A) and end-systole (Figure 1B) phases of the cardiac cycle. 

**Figure 2:**
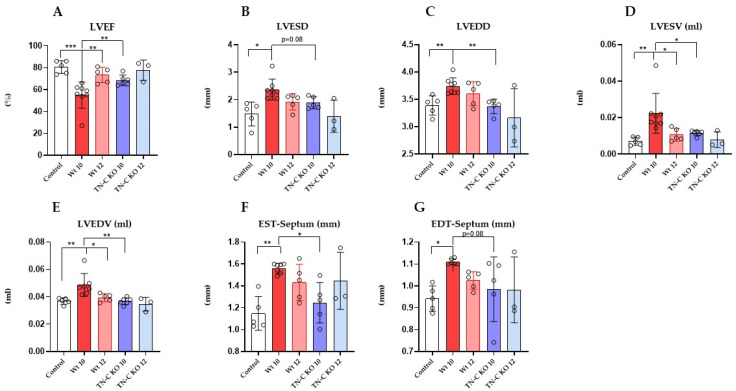
(**A**) Left ventricular ejection fraction (LVEF). (**B**) Left ventricular end-systolic dimension (LVESD). (**C**) Left ventricular end-diastolic dimension (LVEDD). (**D**) Left ventricular end-systolic volume (LVESV). (**E**) Left ventricular end-diastolic volume (LVEDV). (**F**) End-systole septum wall thickness (EST). (**G**) End-diastole septum wall thickness (EDT). * *p* < 0.05, ** *p* < 0.01, *** *p* < 0.001; *n* = 3–8/group.

**Figure 3:**
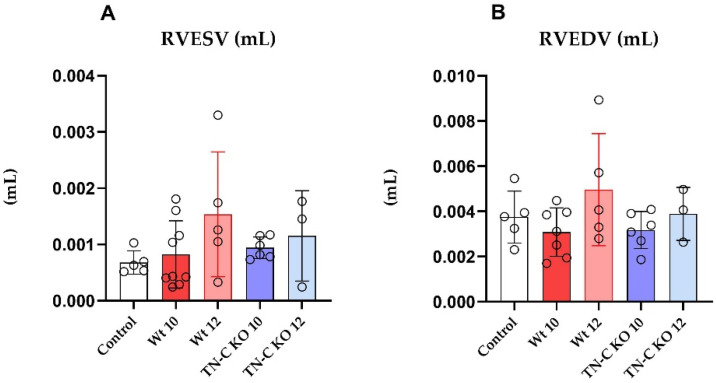
(**A**) Right ventricular end-systolic volume (RVESV) and (**B**) Right ventricular end-diastolic volume (RVEDV) in control, Wt 10, Wt 12, TN-C KO 10, and TN-C KO 12 mice. Wild-Type 10 weeks (Wt 10), Wild-Type 12 weeks (Wt 12), Tenascin-C knockout 10 weeks (TN-C KO 10), and Tenascin-C knockout 12 weeks (TN-C KO 12) mice.

As shown before, in Wt (AJ) mice, 10 weeks of TAC resulted in a significant decrease in the left ventricle ejection fraction (LVEF, Figure 2A; *p* < 0.001) and a significant increase in dimensions (left ventricle end-systolic and diastolic diameter (LVESD and LVEDD), Figure 2B,C; *p* < 0.05 and 0.01, respectively) and volume (LVESV and LVEDV, Figure 2D,E; *p* < 0.01, respectively) as compared to controls. Debanding resulted in a significant improvement in LVEF (Figure 2A; *p* < 0.01) to near-normal levels, as well as the regression of LVEDV and LVESV. In addition, the thickness of the septum (Figure 2F,G) wall significantly increased after 10 weeks of TAC (*p* < 0.01). In contrast, TN-C KO mice after 10 weeks of TAC did not show significantly decreased LVEF nor significantly increased LVEDV and LVESV. Debanding resulted in reconstitution of LVEF (Figure 2A) to near-normal levels as well as the regression of LV volume and dimensions (Figure 2B–E). These findings were paralleled by measurements of LVEDD and LVESD in both Wt and KO mice. Interestingly, we observed a slight increase in right ventricular volume following the debanding procedure in Wt mice (Figure 3A,B). The TN-C KO mice did not show any enlargement with regard to right ventricular volume. 

### 2.3. TN-C Levels and ACE Activity in Cardiac Tissue Samples

**Figure 4:**
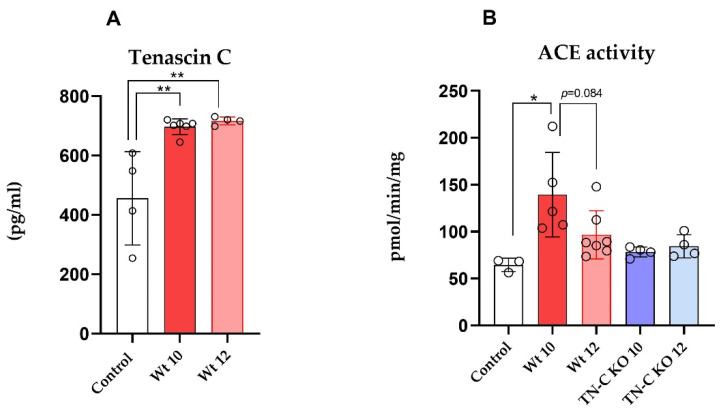
(**A**) Tenascin C (TN-C) levels in left ventricular tissue in control, AJ mice after 10 weeks of banding (Wt 10), and 2 weeks of debanding (Wt 12). (**B**) Angiotensin-converting enzyme (ACE) activity in cardiac tissue in control, Wt, and TN-C KO mice at representative timepoints of banding (10 weeks) and debanding (2 weeks). * *p* < 0.05, ** *p* < 0.01, *n* = 3–7/group. Wild-Type 10 weeks (Wt 10), Wild-Type 12 weeks (Wt 12), Tenascin-C knockout 10 weeks (TN-C KO 10), and Tenascin-C knockout 12 weeks (TN-C KO 12) mice.

As depicted in Figure 4, 10 weeks of TAC significantly increased TN-C levels in LV tissue of WT (AJ) animals (Figure 4A, *p* < 0.01). The debanding procedure, however, did not result in any regression of TN-C expression, indicating continuous pro-fibrotic activation in Wt hearts (Figure 4A). Interestingly, ACE activity was increased in Wt mice following 10 weeks of banding (Figure 4B, *p* < 0.05). However, debanding did not significantly reduce ACE activity in Wt animals in comparison with the Wt 12 group (Figure 4B, *p* = 0.08). This observation was not present in TN-C KO animals, where ACE activity remained unchanged following banding and debanding as compared to control values (Figure 4A,B). 

### 2.4. mRNA Expression of ANP and Collagen 1 (Col 1) in Left Ventricular Tissue Samples

**Figure 5:**
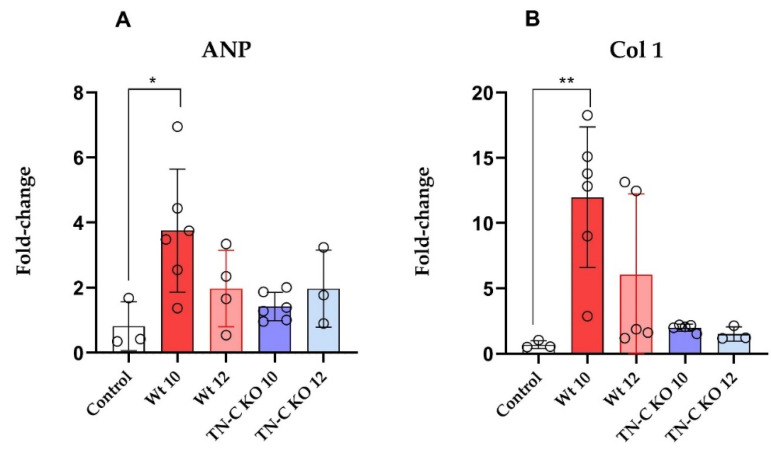
(**A**) Relative fold changes of mRNA expression of atrial natriuretic protein (ANP) and (**B**) collagen 1 (Col 1) between Wt and TN-C KO groups and timepoints (10 and 12 weeks); * *p* < 0.05, ** *p* < 0.01, *n* = 3–7/group. Wild-Type 10 weeks (Wt 10), Wild-Type 12 weeks (Wt 12), Tenascin-C knockout 10 weeks (TN-C KO 10), and Tenascin-C knockout 12 weeks (TN-C KO 12) mice.

As depicted in Figure 5, 10 weeks of TAC significantly increased ANP mRNA expression in cardiac tissue of WT (AJ) animals (Figure 5A,B; *p* < 0.05), an indication of progressive cardiac hypertrophy and dysfunction. The debanding resulted in a regression of ANP, indicating significant unloading of the heart. This observation was paralleled by an increased expression of Col 1, indicating cardiac fibrosis. Debanding resulted in a reduction in Col-1 expression. Although this regression was impressive, the Col-1 expression remained still higher than in the control group. In contrast, in TN-C KO animals, 10 weeks of TAC only slightly changed the mRNA expression of ANP and Col 1, and debanding consequently did not affect these two parameters either, indicating neither progressive heart failure nor fibrosis in these hearts (Figure 5A,B). 

### 2.5. The Effect of TN-C on ACE Expression in Human Ventricular Cardiac Fibroblasts

**Figure 6:**
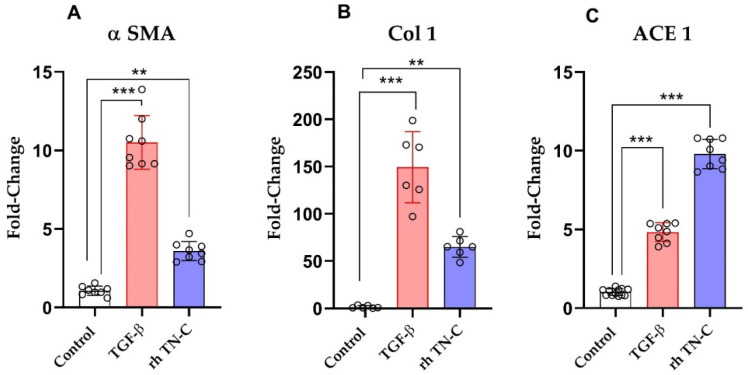
(**A**) Relative fold changes of mRNA expression of alpha-smooth muscle actin (α-SMA), (**B**) collagen 1 (Col 1), and (**C**) angiotensin-converting enzyme (ACE) in human cardiac ventricular fibroblasts after 24 h of incubation with transforming growth factor beta (TGF-β) or recombinant human tenascin-c (rh TN-C). ** *p* < 0.01, *** *p* < 0.001, *n* = 7–8 replicates/group.

As depicted in Figure 6, transforming growth factor beta (TGF-β) administration significantly increased mRNA expression of alpha-smooth muscle actin (α-SMA), Col 1, and ACE-1 (Figure 6A–C; *p* < 0.001, respectively) in human ventricular cardiac fibroblasts, suggesting the causative role in fibrosis. Similarly, human ventricular cardiac fibroblasts treated with recombinant human (rh) TN-C also enhanced the expression of α-SMA and Col 1 (Figure 6A,B; *p* < 0.01, respectively). Notably, the upregulation of ACE 1 was the most significant after rh TN-C application (Figure 6C, *p* < 0.001). 

## 3. Materials and Methods 

### 3.1. Animals

Male TN-C KO mice (KO, RBRC00007 A, Experimental Animal Division, Tsukuba, Japan) and their wild-type littermates (Wt, AJ, #000646, The Jackson Laboratory, Sacramento, CA, USA) were used [13,14,15]. All animals received humane care and were kept under standard laboratory conditions (housed in air-conditioned rooms at 22 °C with a 12/12 h day/night cycle), including free access to water and standard mouse chow. The experimental protocol was approved by the regional Ethics Committee for Laboratory Animal Experiments at the Medical University of Vienna and the Austrian Ministry of Science Research and Economy (BMWFW-66.009/0205-WF/V3b/2015), conforming with the Guide for the Care and Use of Laboratory Animals published by the US National Institutes of Health (NIH Publication No. 85–23, revised 1996).

### 3.2. Experimental Modeling of Cardiac Pressure Overload and Unloading 

The experimental model of LVH progression and regression included both banding and debanding procedures to simulate pressure overload and mechanical unloading, respectively. Mice were briefly anesthetized (1 min) with 4% isoflurane gas and intubated with a 22G peripheral venous catheter for human use (Venflon^®^, B.Braun, Melsungen, Germany) for mechanical ventilation (HSE Minivent 845, Hugo Sachs Electronics, Germany) with 2% isoflurane gas. Animals were placed on a heating plate to maintain body temperature at 37 °C before the extremities were fixed. Eye ointment was applied to prevent dehydration. Buprenorphine (0.1 mg/kg) was administered preoperatively by intraperitoneal injection for analgesia. The chest was shaved and disinfected. A horizontal skin incision (5–10 mm) was performed above the sternum, followed by a minimal hemi-sternotomy in which the upper half of the sternum (about 5 mm) was cut through. The aortic arch was exposed after the thymus had been correctly ousted. TAC surgery was performed by ligating the aorta between the innominate artery and left common carotid artery onto a 27G needle using a 6-0 suture (Prolene^®^, Ethicon, Johnson & Johnson, Somerville, NJ, USA). The cannula ensured the same degree of stenosis for each animal. After successful ligation, the needle was removed, the chest was closed under a positive end-expiratory pressure, and the skin was sutured. Mice were then extubated in recovery of spontaneous breathing and observed postoperatively under a heat lamp. Piritramide in drinking water was applied as a post-operative analgesic regimen (2 ampules of piritramide + 10 mL of 10% glucose in 250 mL water ad libitum). The sham group was operated analogously, except for the aortic ligation. The debanding procedure was performed 10 weeks after the induction of pressure overload. After the incision, the aorta was dissected again to cut through the ligature. Then the chest was closed again and animals received the same postoperative treatment as described above after the first operation and followed for another 2 weeks.

### 3.3. Cardiac Magnetic Resonance Imaging

Magnetic resonance imaging (MRI) was performed for all experimental study groups. In previous studies we demonstrated that there was no difference in baseline cardiac function between Wt (AJ) and TN-C KO mice [10,11]. MRI was performed using a 9.4 Tesla Biospec 94/30 USR system (Bruker Biospin, Ettlingen, Germany). A gradient insert with inner diameter of 116 mm was used. The maximal achievable gradient strength was 667 mT/m. For radiofrequency excitation, a transmitter volume resonator with an inner diameter of 86 mm was used; image acquisition was done using a dedicated mouse heart coil array with 4 elements. Mice were pre-anesthetized with isoflurane 5% and positioned on a dedicated heated mouse bed. Inhaled anesthesia was maintained throughout the examination with a mix of 1.5–2% isoflurane and oxygen via a face mask. During the examination, time respiration, heart rate, and body temperature were monitored. A prospective electrocardiography (ECG)-gated cine gradient echo-based flow-compensated MRI sequence implemented in the in-built software ParaVision 6.0 (Bruker Biospin, Ettlingen, Germany) was acquired in advance in order to visualize cardiac function. A mean of 10 consecutive axial slices along the long axis from the apex to the base of the heart was acquired. The following imaging acquisition parameters were used: time of echo (TE) = 2.4 ms, time of repetition (TR) = 8 ms, averages = 6, field of view (FOV) = 25 mm × 25 mm, slice thickness = 0.8 mm, flip angle = 15°, partial Fourier transformation = 1.45, measured matrix = 132 × 192, visualized matrix = 192 × 192, and 18 movie frames. LV function was assessed using Segment-Software for Quantitative Medical Image Analysis (Segment Software, v1.8 R1172; Medviso AB, Lund, Sweden) [16]. The cine sequence was used to determine the end-systole and end-diastole of the heart action. The end-systolic and end-diastolic volumes (mL) were measured by manual annotation of the left ventricle on each axial slice representing a level along the long axis excluding the papillary muscles. The ejection fraction (%) was calculated automatically. For the LV end-diastolic diameter and LV end-systolic diameter parameters and the measurements of wall thickness, 3–5 independent determinations were done for each mouse and the average was used for calculations. In addition, wall thickness and LV radius were assessed. Figure 1 depicts representative MRI images from left and right ventricle. Supplemental Figure S1 describes the analysis of MRI images following virtual dissection in 6 representative segments. 

### 3.4. Reverse Transcription and Quantitative Polymerase Chain Reaction (PCR)

Total RNA was extracted from left ventricular tissue samples and human ventricular cardiac fibroblasts using the RNeasy Mini Kit (Qiagen, Hilden, Germany) and quantified by nanodrop spectrophotometer. cDNA was prepared using QuantiTect reverse transcription kit (Qiagen, Hilden, Germany). Samples were analyzed in duplicate in a volume of 20 μL per well. The initial denaturation step of 15 min at 95 °C was followed by 45 cycles of 15 s 95 °C, 30 s 50 °C, and 30 s 72 °C, using Rotor-Gene Q (Qiagen, Hilden, Germany) and Rotor-Gene Q series software for Ct value analysis. Relative gene expression was calculated by the 2^−ΔΔ*C*t^ method. The list of primers is depicted in Table S1.

### 3.5. Assessment of TN-C Levles in Cardiac Tissue Samples

A mouse tenascin-C (TN-C) ELISA kit from Cusabio (#CSB-EL023954MO, Wuhan, China) was used. The ELISA kit was used according to the manufacturer’s protocol in order to assess TN-C protein levels in cardiac tissue (left ventricular) samples obtained from sham-operated, aortic banded (10 weeks), and debanded AJ mice. 

### 3.6. Angiotensin-Converting Enzyme Activity Measurement

Angiotensin-converting enzyme (ACE) activity in heart, lung, and kidney tissue samples was measured as originally described [17] and modified [18]. Briefly, tissue samples were weighed, and a proportional amount of 100 mM tris(hydroxymethyl)aminomethane hydrochloride (TRIS) buffer (pH 7.0) was added then homogenized. The tissue homogenates were centrifuged at 13,000 rpm for 5 min and the protein concentration of the supernatant was determined by PierceTM BCA Protein Assay Kit (Thermo Fisher Scientific, Inc., Waltham, MA, USA,) using a TECAN (SparkControl Magellan V2.2, Tecan Systems, Inc., San Jose, CA) plate reader. ACE activity was determined with an artificial substrate (Abz-FRK(Dnp)P-OH (synthesized by Peptide 2.0, Chantilly, VA, USA) in a reaction mixture containing 6 µL of 1 mg/mL tissue homogenates in 35-fold dilution in 100 mM TRIS buffer, 50 mM NaCl, and 10 µM ZnCl_2_. Measurements were performed in 96-well plates (Greiner-Bio One, Vilvoorde, Belgium ) at 37 °C. The fluorescence intensity change was detected by a TECAN (SparkControl Magellan V2.2) plate reader; the λex was 340 nm and λem was 405 nm. The changes in fluorescence intensity were detected in kinetic loops at 1-min intervals for at least 30 min and the intensity values were plotted as a function of reaction time. The fluorescence intensity values were fitted by a linear regression (GraphPad Software, San Diego, CA, USA), and the fit with the data was accepted only when the r^2^ was >0.9. ACE activity was calculated by the following equation: activity = (S/k) × D/P; where S is the rate of the increase in fluorescence intensity (1/min), k is the change of fluorescence intensity during the complete cleavage of 1 pmol Abz-FRK(Dnp)P-OH substrate, D is the dilution of the sample, and P is the mg/mL protein concentration. One unit (U) indicates 1 pmol substrate cleavage in 1 min by 1 mg of protein.

### 3.7. Human Ventricular Cardiac Fibroblast Experiments

Human ventricular cardiac fibroblasts (Lonza, Basel, Switzerland) were cultured in fibroblast basal medium supplemented with 0.1% insulin, 0.1% fibroblast growth factor, 0.1% GA-1000, and 10% FBS (all Lonza, Basel, Switzerland). Cultures were washed with HEPES buffered saline (Lonza, Basel, Switzerland) when indicated, and split at a confluency level of 70%. Cells were serum starved for 24 h, with subsequent treatment with one of the following: (1) No treatment—control; (2) 20 ng/mL TGFβ (Abcam, Cambridge, UK); or (3) recombinant human tenascin C (1 µg/mL rh TN-C, Fisher Scientific, Waltham, MA, USA) for an additional 24 h. Then, the total RNA was extracted as described previously [19]. 

### 3.8. Statistical Analysis

All results are expressed as mean ± standard deviation (SD). Comparisons among groups were analyzed by nonparametric one-way analysis of variance (ANOVA) with a post hoc Turkey’s test. Comparisons between the corresponding banding–debanding mice (AJ vs. TNC knockout groups) were made using an unpaired *t*-test. Prism^TM^9 software (GraphPad Inc., San Diego, CA, USA) was used for all statistical analysis. *p* values < 0.05 were considered significant.

## 4. Discussion

The present experimental study describes for the first time the effects of 10 weeks of banding followed by 2 weeks of debanding and highlights the effects of TN-C. In Wt animals, banding led to significant deterioration of cardiac function and LV and RV dilatation. This was paralleled by increased expression of markers of heart failure and fibrosis. Debanding of these failing hearts led to reverse remodeling, as indicated by partial reduction of ACE-1 activity and ANP and Col 1 expression. TN-C expression in Wt hearts was significantly upregulated after 10 weeks of banding and remained unchanged after debanding. TN-C KO mice did not show a significant reduction in LVEF nor dilatation of the left and right ventricles. In fact, debanding did not result in significant functional improvements. In TN-C KO mice we found no evidence of increased ACE activity, nor progression of heart failure or fibrosis. In human ventricular cardiac fibroblasts, rh TN-C resulted in significant upregulation of ACE-1 expression but also of Col 1 and α-SMA, all indicating the importance of TN-C in activation and preservation of the profibrotic stimulus under these experimental conditions of pressure overload.

The present observations are in accordance with our recent findings in the same model of TAC [10]. We were able to show that after 10 weeks of banding, TN-C KO animals showed preserved LV function and less LV hypertrophy. We speculated that TN-C-mediated signaling pathways in the progression of LVH under pressure overload may provide a therapeutic target to improve cardiac function in patients with LVH and heart failure. In the present study we expanded our former observations by a period of 2 weeks of debanding. Thereby, we wanted to simulate the clinical scenario of aortic valve replacement in aortic valve stenosis. Bjornstad et al. [7] recently demonstrated that after 4 weeks of banding and consecutive debanding (14 days), a progressive and complete improvement of function and regression of myocardial mRNA levels of BNP, ANP, α-SMA were observed. While the data of Bjornstad et al. [7] represent the state of compensated hypertrophy, our study design went beyond this to assess decompensated hypertrophy or failure. The latter is indicated by the reduction in EF in these mice. In human hearts, the physiological EF is around 50–55%, and a reduction to 35–40% is considered as severe impairment; in mice, physiological EF is beyond 80%. After 10 weeks of banding in Wt10, EF is significantly reduced to 50% and increased lung weight suggests pulmonary congestion, both indicating a state of decompensation. In order to find out more about the role of ECM remodeling and in particular about TN-C, we studied the same protocol in TN-C KO mice. Consequently, our data describe how after 10 weeks of pressure overload, debanding in TN-C KO mice resulted in complete restoration of LVEF and remodeling of the left and right ventricles. Debanding also resulted in a reduction in ACE 1 activity. This finding is underpinned by the fact that debanding in Wt did not affect myocardial TN-C expression. It is therefore reasonable to speculate that TN-C is one of the key-players in the progression of LV dilatation and fibrosis and TN-C directly activates ACE 1 activity, as depicted by our in vitro experiments in human cardiac fibroblasts. 

In an experimental model of myocardial infarction in TN-C KO we recently showed that cardiac output and external heart work were elevated, while LV wall stress and collagen expression were decreased as compared to age-matched controls [11]. Similar to the present results, ACE 1 activity in the peri-infarct zone was also significantly lower in TN-C KO animals, leading to less fibrosis and LV dilatation.

Clinically, the most important question is with regard to when and whether LV remodeling reversal occurs. In the setting of compensated hypertrophy with preserved LVEF and aortic valve stenosis, Kreyenbuehel et al. described reverse remodeling in early, intermediate, and late phases after replacement [2] in a cohort of 27 patients. The major finding was that in these patients, regression of interstitial fibrosis can occur late (60 months) after surgery. In the recently published Foundation and Triton trials in a similar cohort of patients with normal LVEF undergoing aortic valve replacement using a newly designed pericardial valve, the authors were able to show a reduction in transvalvular mean pressure gradient from 47.6 to 9.6 mm Hg at one year after surgery [20], and a persistent regression of LVM (index) using transthoracic echocardiography over 5 years [21]. It therefore seems that patients with preserved LVEF show persistent reverse remodeling following aortic valve replacement. In a completely different cohort of patients with highly reduced LV function and heart failure undergoing LV assist device implantation, unloading of the heart did not induce reverse remodeling [6]. In contrast, cardiac fibrosis further increased, as indicated by 4-hydroxyproline content [7]. Therefore, it seems reasonable to speculate that reverse remodeling is challenging in decompensated hypertrophy and failure. Our current experimental findings underline these clinical observations. In the Wt group, 10 weeks of banding and 2 weeks of debanding led to moderate functional reverse remodeling and partial but not significant reduction of ACE 1 activity. This finding is particularly important in the clinical scenario and for confirming the rational use of ACE inhibitor or Ang II receptor blockers following elective aortic valve replacement surgery. Magne et al. [22], in an observational study, demonstrated that the use of these inhibitors was associated with improved long-term outcomes after isolated surgical aortic valve replacement. Although we do not have experimental evidence due to a lack of spatiotemporal inhibition of TN-C, it is tempting to speculate that remaining higher TN-C expression after debanding surgery may contribute to and explain higher ACE activity in cardiac tissue as compared to control hearts. In a manner similar to reparative fibrosis after infarction, various external stimuli such as pressure overload activate cardiac fibroblasts to acquire the myofibroblast phenotype and synthesize collagens. The activation of myofibroblasts is dependent on the recruitment of monocyte-derived macrophages originating from the bone marrow [23]. They produce IL-6, which, in turn, activates the TGF-β1/Smad pathway, a well-known profibrotic cascade [24]. 

A recent publication by Perestrelo et al. [25] added additional information to the puzzle of ECM remodeling. In their clinical and experimental decellularized samples of failing myocardium, they identified a common pattern of aligned, flat, and compact fiber bundles with reduced elasticity and organizational complexity. At the molecular level, RNA sequencing of HF cardiac fibroblasts highlighted the overrepresentation of dysregulated genes involved in ECM organization or a connection to TGF-β1, interleukin-1, TNF-α, and Brain-Derived Neurotrophic Factor (BDNF) signaling pathways. Again, in our own ex vivo analysis in human cardiac fibroblasts we were able to show that TGF-β1, a well described inducer of cardiac fibrosis, upregulated α-SMA, ACE 1, and Col 1 expression. These newly described patterns of fiber bundles in failing hearts must be kept in mind as structures that scarcely change following unloading and that are probably responsible for the “fixation” or even progression of fibrosis in failing hearts. 

The clinical relevance of TN-C upregulation has been demonstrated in patients with dilated cardiomyopathy. Yokokawa et al. [26] assigned 123 patients to high- or low-level TN-C groups according to tissue TN-C expression in right ventricular biopsies. High levels were associated with diabetes mellitus, progressive LV dilatation, and poorer survival. The authors concluded that TN-C was an independent predictor for poor outcome. Similarly, serum levels of TN-C were elevated in patients with HF and correlated nicely with LV dysfunction and 12-month major adverse cardiac events [27]. In patients with coronary artery disease, the degree of artheriosclerosis correlates with serum TN-C levels [28]. Of importance, patients with high serum levels of TN-C during the acute stage after myocardial infarction are at a higher risk of ventricular dilatation several months later and show worse long-term prognoses [29,30], suggesting the involvement of TN-C in the progression of post MI adverse ventricular remodeling.

The current study has the following limitations. First, the number of mice in this longitudinal study that finished the complete follow-up of 12 weeks (including two surgical interventions and two MRI studies) was lower than we intended. It should be stressed that the mice underwent four anesthetic procedures with two complex surgeries and repetitive blood losses. However, a consecutive readout of data was possible, with a low number of outliers. Therefore, we believe that we can present reproducible data to our readers. Second, we used full-body TN-C KO (not conditional KO); we did not use si-RNA-TN-C or TN-C antibodies (lacking specificity and sensitivity) to address the lack of TN-C in the reverse remodeling phase. Therefore, our results do not allow any conclusions on the potential timing of future TN-C related interventions and we can only speculate on the causative role of TN-C in incomplete reverse remodeling. Indeed, mechanical unloading (debanding) mitigates pressure overload-induced mechanical stretch, which plays a dominant role in ACE upregulation. This latter effect results at least in partial ACE downregulation. Nevertheless, our study not only shows the association between TN-C and ACE but also provides evidence that TN-C stimulates ACE expression in cardiac fibroblasts. In line with this, TN-C KO mice showed less upregulation of ACE activity in cardiac tissue. Third, we did not use late gadolinium enhancement on cardiac magnetic resonance nor histological staining to measure cardiac fibrosis upon reverse remodeling. Finally, the expression of ANP, Col 1, α-SMA, and ACE-1 was presented only at mRNA level. These data were derived either from limited amounts of mouse tissue or cell culture. They are in good correlation with already published protein data from our own group and others. 

## 5. Conclusions

In summary, we can conclude that pressure overload, when lasting long enough to induce HF, has less potential for reverse remodeling. This phenomenon is associated with a significant upregulation of TN-C expression, which stimulates ACE-1, Col 1, and α SMA upregulation in cardiac fibroblasts. Consequently, addressing TN-C in LV hypertrophy may open a new window for future therapeutics. 

## Figures and Tables

**Table 1:** Animal characteristics from each group: Controls, Wild-Type 10 weeks (Wt 10), Wild-Type 12 weeks (Wt 12), Tenascin-C knockout 10 weeks (TN-C KO 10), and Tenascin-C knockout 12 weeks (TN-C KO 12) mice.

	BW (g)	HW (g)	HW/BW (×1000)	LVM (g)	LVM/BW (×1000)	LW/BW (×1000)
Controls	24.82 ± 2.68	0.11 ± 0.02	4.6 ± 0.04	0.069 ± 0.006	2.72 ± 0.15	51 ± 3
Wt 10	28.33 ± 1.90	0.19 ± 0.03 ***	6.5 ± 0.01 **	0.097 ± 0.011 **	3.84 ± 1.10 **	90 ± 21 **
Wt 12	26.74 ± 0.96	0.14 ± 0.02 ##	5.2 ± 0.05 ##	0.074 ± 0.006 ##	2.82 ± 0.20 ##	61 ± 28
TN-C KO 10	26.68 ± 1.75	0.14 ± 0.03	5.0 ± 0.07	0.074 ± 0.011	2.85 ± 0.67	50 ± 9
TN-C KO 12	26.54 ± 0.92	0.12 ± 0.01	5.1 ± 0.05	0.065 ± 0.011	2.54 ± 0.25	51 ± 5

Statistical differences are indicated with asterisks (*** for *p* < 0.001, and ** for *p* < 0.01) between controls and 10-week banding groups and ## for *p* < 0.01 between Wt 10 and Wt 12 groups. BW: body weight; HW: heart weight; LVM: left ventricular mass, LW: wet lung weight.

## Data Availability

Data is contained within the article or supplementary material.

## Supplementary Materials

The following are available online at https://www.mdpi.com/1422-0067/22/4/2023/s1. Figure S1: Graphical representation of the segmentation analysis for the short-axis MRI, at the mid-left ventricular slice (Left panel). Once the left ventricular endocardium and LV epicardium were outlined in red and green (respectively), the myocardium wall thickness and ventricular radius were analyzed in 6 different segments (right panel). Table S1: List of primer sequences.

## Abbreviations

α-SMA	Alpha smooth muscle actin
ACE	Angiotensin-converting enzyme
Ang II	Angiotensin II
BDNF	Brain-Derived Neurotrophic Factor
BNP	Brain natriuretic peptide
BP	Blood pressure
BW	Body weight
Col 1	Collagen I
ECM	Extracellular matrix
EDV	End diastolic volume
EF	Ejection fraction
ESV	End systolic volume
FOV	Field of view
HCF	Human cardiac fibroblast
HW	Heart weight
LV	Left ventricular
LVEDD	Left ventricular end-diastolic diameter
LVEDV	Left ventricular end-diastolic volume
LVEF	Left ventricular ejection fraction
LVESD	Left ventricular end-systolic diameter
LVESV	Left Ventricular end-systolic volume
LVH	Left ventricular hypertrophy
LVM	Left ventricular mass
LW	Lung wet weight
MRI	Magnetic resonance imaging
RV	Right ventricular
RVEDV	Right ventricular end-diastolic volume
RVESV	Right ventricular end-systolic volume
RVM	Right ventricular mass
TAC	Transverse aortic constriction
TE	Echo time
TGF-β	Transforming growth factor beta
TN-C	Tenascin C
