# Supplementary material for: The Role of Tenascin C in Cardiac Reverse Remodeling Following Banding–Debanding of the Ascending Aorta
Source: Int J Mol Sci. 2021 Feb 18;22(4):2023. doi: 10.3390/ijms22042023 (PMC7921966; doi:10.3390/ijms22042023)
Supplement: Supplementary file 1 [file ijms-22-02023-s001.pdf]

Supplementary Materials:

Table S1. List of primers sequences.

	Gene	Forward 5′-3′	Primer lenght (bp)	Reverse 5′–3′	Primer lenght (bp)	Product (bp)
mouse	GAPDH	GCACCGTCAAGGCTGAGAACC	20	ATGGTGGTGAAGACGCCAGT	20	94
	Col 1	GGAGAGTACTGGATCGACCCTAAC	25	CTGACCTGTCTCCATGTTGCA	21	100
	ANP	CAAGAACCTGCTAGACCACC	20	AGCTGTTGCAGCCTAGTCC	20	96
human	S18	GTAACCCGTTGAACCCCATT	20	CCATCCAATCGGTAGTAGCG	21	112
	ACE 1	ACAGGTGCTGTTCCAGAGCG	20	TGCGGGTCCGTGAAGTTCTG	20	174
	α-SMA	GCAAACTGAAAGTTGGCTCC	20	ATCGTGTTGAATTCTGAGGCA	21	100
	Col 1	TGTTTGGGTCATTTCCACATGC	22	AGCACAAAGCAGTTTTTCCCC	21	135

GAPDH: Glyceraldehyde 3-phosphate dehydrogenase, Col 1: collagen 1, ANP: atrial natriuretic, S18: 18S ribosomal RNA peptide, ACE1: angiotensin converting enzyme 1, α-SMA: alpha smooth muscle actin

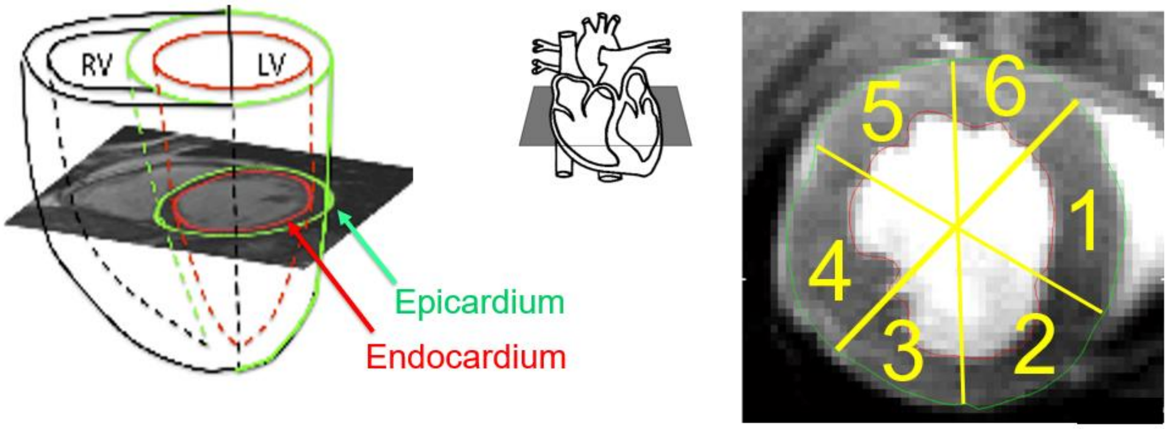

Figure S1. Graphical representation of the segmentation analysis for the short-axis MRI, at the mid-left ventricular slice (Left panel). Once the left ventricular endocardium and LV epicardium were outlined in red and green (respectively), the myocardium wall thickness, and ventricular radius were analyzed at 6 different segments (right panel).
